# Supplementary figures and images for: Sexually transmitted infections among key populations in India: A protocol for systematic review
Source: PLoS One. 2023 Mar 13;18(3):e0279048. doi: 10.1371/journal.pone.0279048 (PMC10010531; doi:10.1371/journal.pone.0279048)

**SUPPORTING FILE NO-02**

**Data Extraction Form**


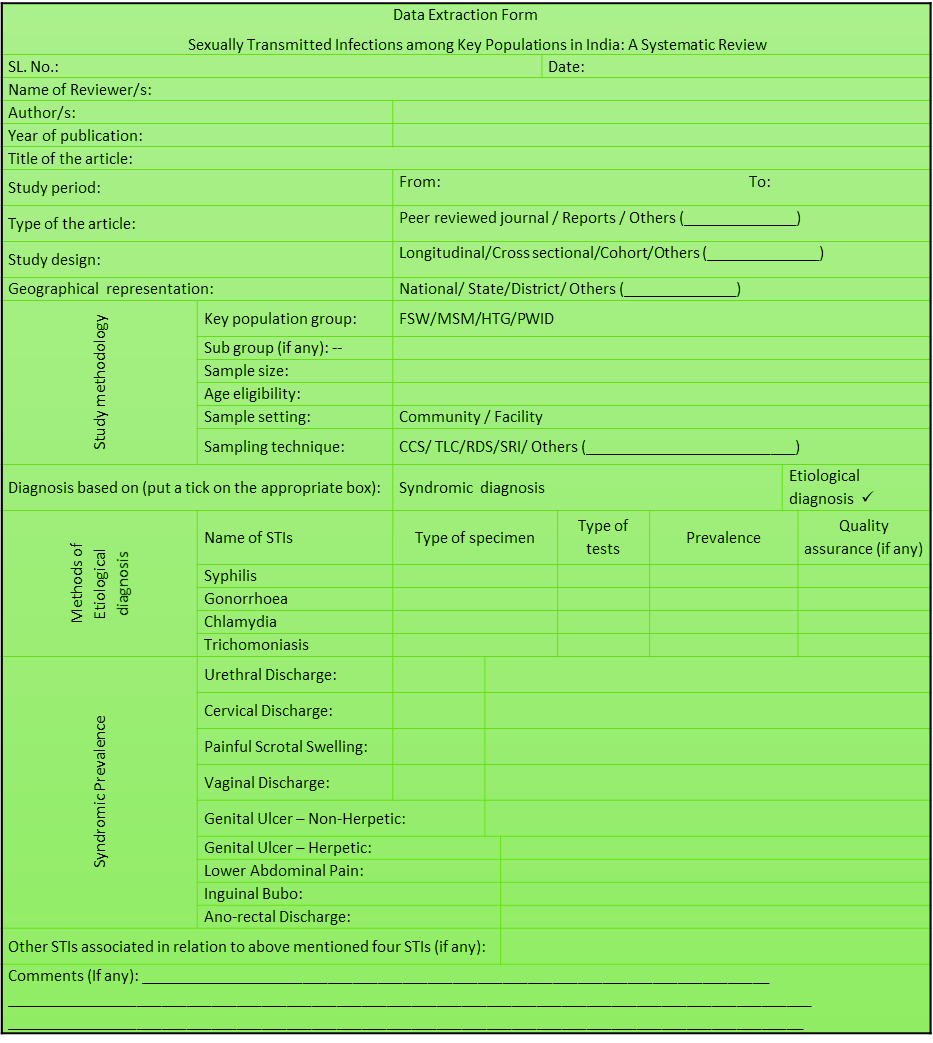

Supplement: S2 File — (DOCX) [file pone.0279048.s003.docx]
